# A nature-based approach for managing the invasive weed species *Gutenbergia cordifolia* for sustainable rangeland management

**DOI:** 10.1186/s40064-016-3480-y

**Published:** 2016-10-13

**Authors:** Issakwisa B. Ngondya, Linus K. Munishi, Anna C. Treydte, Patrick A. Ndakidemi

**Affiliations:** 1 Department of Sustainable Agriculture, Biodiversity and Ecosystem Management, School of Life Sciences and Bio-Engineering, The Nelson Mandela African Institution of Science and Technology, P.O. Box 447, Arusha, Tanzania; 2 Ministry of Natural Resources and Tourism, Wildlife Division, Tourism Hunting, Photographic Tourism and CITES Office, P.O. Box 1541, Arusha, Tanzania

**Keywords:** Allelopathy, Bioherbicide, Desmodium, Ecological invasions, Ngorongoro

## Abstract

**Background:**

The invasive weed species *Gutenbergia cordifolia* has been observed to suppress native plants and to dominate more than half of the entire crater floor (250 km^2^) in the Ngorongoro Conservation Area (NCA). As this species has been found to be toxic to ruminants it might strongly impact animal populations in this ecologically diverse ecosystem. Hence, a nature-based approach is urgently needed to manage its spread. We tested two *Desmodium spp* extracts applied to *G. cordifolia* and assessed the latter’s germination rate, height, fresh weight and leaf total chlorophyll content after 30 days in both laboratory and screen house experiments.

**Results:**

Seedling germination rate was halved by *Desmodium uncinatum* leaf extract (DuL), particularly under higher concentrations (≥75 %) rather than lower concentrations (≤62.5 %). Likewise, in both laboratory and screen house experiments, germination rate under DuL treatments declined with increasing concentrations. Seedling height, fresh weight and leaf total chlorophyll content (Chl) were also most strongly affected by DuL treatments rather than *D. uncinatum* root extract, *Desmodium intortum* leaf extract or *D. intortum* root extract treatments. Generally, seedlings treated with higher DuL concentrations were half as tall, had one-third the weight and half the leaf Chl content compared to those treated with lower concentrations.

**Conclusion:**

Our study shows a novel technique that can be applied where *G. cordifolia* may be driving native flora and fauna to local extinction. Our data further suggest that this innovative approach is both ecologically safe and effective and that *D. uncinatum* can be sustainably used to manage invasive plants, and thus, to improve rangeland productivity.

## Background

The management of invasive plant species in protected areas has posed a lot of challenges to both managers and conservationists, due to possible negative effects of herbicides on other native plant species (Poorter et al. 2007). While methods such as mechanical uprooting can be applied, they are only a short term remedy as a lot of invasive plant seeds remain in the soil seedbank. Moreover, this method often results in high operational costs and is, therefore, economically unfeasible. *Gutenbergia cordifolia* is an unpalatable invasive plant and can result in both allergies and toxicity to animals as it contains germacranolides (Zdero and Bohlmann 1990; Bussmann et al. 2006). In East Africa, this plant has already been reported as an invasive weed in most farmlands (Anderson and Morales 2005; Gharabadiyan et al. 2012). In Tanzania, in 1962 this plant species dominated in several square miles of the Ngorongoro Crater in the Ngorongoro Conservation Area (NCA) (Beentje 2000) but it rapidly increased and now dominates over a half of the entire crater floor (250 km^2^) (UNESCO 2001). In the Ngorongoro crater, the invaded areas do not appear to be utilized by animals, likely due to a lack of palatable forage species (pers. obs.). If *G. cordifolia* is left unmanaged it is increasingly likely that there will be an ongoing loss of forage resources and habitat for wildlife. This plant species might spread further to other protected areas (e.g. Serengeti National Park, Lake Manyara National Park) where the management could be difficult as the use of herbicides is strictly prohibited.

While allelopathic woody plants in forests have been intensively studied, considerably less research has been conducted on allelopathic herbaceous angiosperms (Rice 1979). In NCA, an important factor contributing to the decline of animal populations, particularly that of ungulates, is the replacement of palatable plants by less-palatable grasses and weeds such as *G. cordifolia* (Estes et al. 2006). The roots of *G. cordifolia* produce allelochemicals, as many invasive plants do, which delay native plant germination and, thus, reduces native forage available for wildlife (Li et al. 2010). Generally, allelopathic plants have various effects on their local neighbours, from inhibition of nodulation in legumes (Murthy and Ravindra 1974; Rice 1979) to reducing chlorophyll production and inhibiting respiratory plant activity (Reza 2016).

Mechanical removal, traditional and chemical controls have been recommended to be applied whenever an invasive weed invades an area (Mada et al. 2013). However, as chemical control using herbicides is often not advised in natural ecosystems, and particularly in protected rangelands, less ecologically intrusive management options must be adopted. Hence, recently, the possibility of using native plants with allelopathic properties as a potential bioherbicide has been discussed (Khanh et al. 2007; Sodaeizadeh and Hosseini 2012; Khan et al. 2008; Ngondya et al. 2016).

Successful management of a particular weed strongly depends on the available knowledge of its life history. While the medicinal use of *G. cordifolia* has been studied (Koch et al. 2005; Ngezahayo et al. 2015) little is known about its germination and growth characteristics as a pre-caution for its control once it escapes as a weed, especially in protected areas. We, therefore, chose to investigate the allelopathic effects of leaf and root crude extracts of two commonly known allelopathic species (*Desmodium uncinatum* and *Desmodium intortum*) (Krishnamurthy et al. 2011; Pickett et al. 2013) on the germination and growth characteristics of the invasive *G. cordifolia*. We chose these two *Desmodium* species as they are generally preferred by herbivores (Heuze et al. 2015a, b) and, thus, could be inter-planted in invaded areas to potentially suppress *G. cordifolia.* Further, these two species successfully suppressed germination of the weed plant *T. minuta* (Ngondya et al. 2016) and, hence, we expected the same effect towards *G. cordifolia*. While providing feed to animals, particularly *D. uncinatum* is known to successfully control some very problematic weeds such as *Striga* species (Khan et al. 2006a, b; Pickett et al. 2013). The mechanism behind *D. uncinatum* allelopathic nature is reportedly due to its high content of isoflavonoids, which have recently been reported to inhibit the growth of other plants (Khan et al. 2008). However, no efforts have been done to study the effects of *D. uncinatum* and *D. intortum* root and leaf crude extracts on germination and growth of *G. cordifolia,* which is of particular interest as a pre-requisite in devising a management option to suppress *G. cordifolia* in nature reserves. Thus, using extracts from these species might be a highly successful, eco-friendly and cheap management tool. We tested the effects of root and leaf crude extracts of *D. uncinatum* and *D. intortum* on the seed germination, seedling height, seedling fresh weight and leaf total Chlorophyll content of *G. cordifolia*, which are important measures of seedling vigor.

## Methods

### Laboratory study design

The effects of *D. uncinatum* and *D. intortum* leaf and root crude extracts on the seed germination, seedling height, leaf chlorophyll content and fresh weight (biomass) of *G. cordifolia* were studied using a completely randomized design from October to November 2015. Ten seeds of *G. cordifolia* were placed in each of six petri dishes (70.84 cm^2^ surface area) lined with cotton wool, and subjected to six different concentration treatments, each treatment was replicated three times, which summed up to 72 samples overall. Distilled water was added ad libitum to moisten the seeds. Seeds were observed every day and the number of germinated seeds were recorded and counted for 30 days. After 30 days, seedlings were harvested and fresh weight, seedling height and leaf total chlorophyll content were determined for each germinated seedling.

### Screen house study design

The effects of leaf and root crude extracts of *D. uncinatum* and *D. intortum* on the seed germination, seedling height, leaf total chlorophyll content and fresh weight of *G. cordifolia* were studied using a completely randomized design in a screen house from October to November 2015. Ten seeds of *G. cordifolia* were placed in each of six pots (763.82 cm^2^ surface area) under six different concentration treatments. Each treatment was replicated three times (n = 72). Normal tap water was added ad libitum. Seeds were observed every day and the number of germinated seeds were recorded and counted for 30 days. After 30 days, seedlings were harvested and fresh weight, seedling height and leaf total chlorophyll content were determined for each germinated seedling.

### Root and leaf crude extract preparation

Fresh roots and leaves from young *D. uncinatum* and *D. intortum* were collected from the Livestock Training Institute (LITI), Tengeru demonstration plots, in early January 2015. Roots and leaves were air dried under room temperature for 14 days, ground into powder and stored in sealed plastic bags prior to experiment. Extracts were prepared according to Namkeleja et al. (2014) as follows: for each species, 100 g of root and leaf powder were soaked separately in 1 l of distilled water and left for 72 h, after which the crude extracts were filtered using Watsman filter paper No. 1 to obtain a final volume of 1 l each. Both crude extracts (ml) were diluted with distilled water (ml) in the ratio of; 0:100, 25:75, 50:50, 62.5:37.5, 75:25 and 100:0 (extract: distilled water) to obtain different concentrations (100 ml each) of 0, 25, 50, 62.5, 75 and 100 %. The diluted extracts were stored at 4 °C prior to experiment.

### *G. cordifolia* seed preparation and treatment

Seeds of *G. cordifolia* were collected from Ngorongoro Crater in late August 2015. Prior to the experiment, the seeds were air dried and stored in plastic bags. *G. cordifolia* seed viability was determined by germination testing (Wildfong 2014), in which all fifteen seeds (100 %) that were selected randomly from a seed stock and planted in a petri dish lined with cotton wool in early September 2015, germinated. Seeds were washed using tap water and sterilized with 5 % NaOCl for 2 min then rinsed with distilled water before planting. Each petri dish/pot was irrigated once with 10 ml/100 ml respectively, of the different solution treatments, i.e., T_1_ = 25 %, T_2_ = 50 %, T_3_ = 62.5 %, T_4_ = 75 % and T_5_ = 100 %. The seeds that were treated with distilled water only (T_0_ = 0 %) were taken as a control.

### Leaf total chlorophyll determination

Leaf chlorophyll of the *G. cordifolia* seedlings was extracted according to Hiscox and Israelstam (1978) with some modifications: 50 mg of *G. cordifolia* fresh leaves of 2.25 cm^2^ surface area were immersed in 4 ml of Dimethyl Sulfoxide (DMSO) and incubated at 65 °C for 12 h. The extract was transferred to glass cuvettes for absorbance determination. The absorbance of blank liquid (DMSO) and samples were determined under 2000 UV/VIS spectrophotometer (UNICO^®^) at 645 and 663 nm (Hiscox and Israelstam 1978) and the leaf total chlorophyll content (Chl) calculated according to Arnon (1949) using the following equation:

$$Total\,Chl = 0.0202\,A_{663} + 0.00802\,A_{645}$$

where *A*_*663*_ and *A*_*645*_ are absorbance readings at 663 and 645 nm, respectively.

### Statistical analysis

Shapiro–Wilk test for normality was performed on seedling height, fresh weight and Chl contents of *G. cordifolia* under all treatment types and levels (DuL, DuR, DiL and DiR) in both, laboratory and screen house experiments. For all data that passed normality test, one-way analysis of variance (ANOVA) was carried out whilst for non-normally distributed data, a Kruskal–Wallis test was performed using STATISTICA version 8 (StatSoft Inc. 2007). The resulting means were separated by the Fisher’s Least Significant Difference (LSD) test at *p* = 0.05. One-way ANOVA was performed on seedling heights and fresh weight under DuL, DuR, DiL and DiR treatments, and Chl contents under DuR, DiL and DiR, while Kruskal–Wallis test was performed on Chl contents under DuL. Generalized linear models were performed on the data using R- software version 3.3.1 (R-Core Team 2016), whereby simple linear regression analysis was done to predict the effects of DuL, DuR, DiL and DiR extracts on seedling height, fresh weight and Chl content while logistic regression (logit) models were performed to predict seedling germination rate under varying root and shoot extract concentration. Germination was considered as a binary response (dependent) variable where 1 denoted as seed was “germinated” and 0 “not germinated”, while extract concentration and plant parts (leaf and root extracts) were independent variables.

## Results

### Allelopathic effects on seed germination

Germination data was calculated and summarized as mean percent germination (Tables 1, 2). In both laboratory and screen house experiments higher concentrations (≥75 %) were more effective in suppressing germination than lower concentrations. A 100 % DuL extract was observed to be the most effective, with as much as three times the suppressive effect of DuR, DiL and DiR (Tables 1, 2).

**Table 1:** Mean (±SE) percentage germination of *G. cordifolia* seeds per treatment of *D. uncinatum* and *D. intortum* leaf and root extracts in different concentrations after 30 days of treatment in the laboratory

Concentration (%)	*D. uncinatum*		*D. intortum*	
	Leaves	Roots	Leaves	Roots
0.0	87 ± 3	83 ± 9	73 ± 2	83 ± 9
25.0	83 ± 9	80 ± 1	76 ± 3	77 ± 3
50.0	57 ± 4	77 ± 3	73 ± 3	70 ± 9
62.5	67 ± 9	77 ± 9	83 ± 7	60 ± 9
75.0	40 ± 0	70 ± 5	70 ± 0	80 ± 9
100.0	33 ± 3	63 ± 7	67 ± 7	73 ± 3

**Table 2:** Mean (±S.E) percentage germination of *G. cordifolia* seeds per treatment of *D. uncinatum* and *D. intortum* leaf and root extracts in different concentrations after 30 days of treatment in the screen house experiment

Concentration (%)	*D. uncinatum*		*D. intortum*	
	Leaves	Roots	Leaves	Roots
0.0	97 ± 0.2	90 ± 0.2	92 ± 0.2	82 ± 0.2
25.0	63 ± 0.2	80 ± 0.2	86 ± 0.2	88 ± 0.2
50.0	60 ± 0.1	90 ± 0.2	75 ± 0.2	72 ± 0.2
62.5	57 ± 0.1	87 ± 0.2	66 ± 0.2	81 ± 0.2
75.0	50 ± 0.1	73 ± 0.2	72 ± 0.2	80 ± 0.2
100.0	40 ± 0.1	76 ± 0.2	82 ± 0.2	86 ± 0.2

In both laboratory and screen house experiments, the results indicated that for every one unit of DuL extract concentration increase the seed germination rate (germinated versus un-germinated) decreased by 0.012 and 0.001, respectively (Table 3).

**Table 3:** Logistic regression analysis on the effect of extracts on *G. cordifolia* seed germination after 30 days in the laboratory and screen house experiments

Extract type	Laboratory				Screen house			
	Estimate	*S.E*	*Z*	*p*	Estimate	*S.E*	*Z*	*p*
Intercept	1.253	0.2193	5.714	1.1 × 10^−8^	0.713	0.0419	16.990	2 × 10^−16^
DuL	−0.012	0.0027	−4.496	6.9 × 10^−6^	−0.001	0.0005	−3.593	3.5 × 10^−4^
DuR	0.519	0.2358	2.205	0.027	0.211	0.0437	4.836	1.6 × 10^−6^
DiL	0.459	0.2340	1.965	0.049	0.088	0.0519	1.700	0.089
DiR	0.582	0.2378	2.447	0.014	0.206	0.0437	4.709	2.9 × 10^−6^

#### Allelopathic effects on plant characteristics

Seedling height differed significantly for DuL, DuR and DiL treatments (*p* < 0.05) but not in DiR in the laboratory experiment (Table 4).

**Table 4:** Kruskal-Wallis and one-way ANOVA test of *G. cordifolia* seedling parameters (mean seedling fresh weight, height and leaf total chlorophyll content) per treatment after 30 days of treatment in a laboratory experiment (*H* = *H*_*(5,18)*_ and *F* = *F*_*(5,18)*_)

Parameters	*D. uncinatum*		*D. intortum*	
	Leaves	Roots	Leaves	Roots
Seedling height	*F* = 9.40***	*F* = 4.52*	*F* = 6.66**	*F* = 1.82
Fresh weight	*F* = 12.97***	*H* = 3.43	*F* = 4.75*	*F* = 1.84
Chl	*F* = 44.38****	*H* = 9.02	*F* = 1.72	*F* = 11.45***

* *p* < 0.05; ** *p* ≤ 0.01; *** *p* ≤ 0.001; **** *p* ≤ 0.0001

In the screen house experiment, seedling height differed significantly only under DuL and DiL treatments (*p* < *0.05*) (Table 5).

**Table 5:** Kruskal-Wallis and One-way ANOVA test of *G. cordifolia* seedling parameters (height, fresh weight and leaf total chlorophyll content (Chl)) per treatment after 30 days of treatment in a screen house experiment (*H* = *H*_*(5,18)*_ and *F* = *F*_*(5,18)*_)

Parameters	*D. uncinatum*		*D. intortum*	
	Leaves	Roots	Leaves	Roots
Seedling height	*F* = 22.21****	*F* = 2.22	*F* = 6.17**	*F* = 0.15
Fresh weight	*F* = 11.56***	*F* = 0.97	*F* = 1.21	*F* = 5.35**
Chl	*H* = 13.96*	*F* = 7.66**	*F* = 1.58	*F* = 12.52***

* *p* < 0.05; ** *p* ≤ 0.01; *** *p* ≤ 0.001; **** *p* ≤ 0.0001

**Fig. 1:**
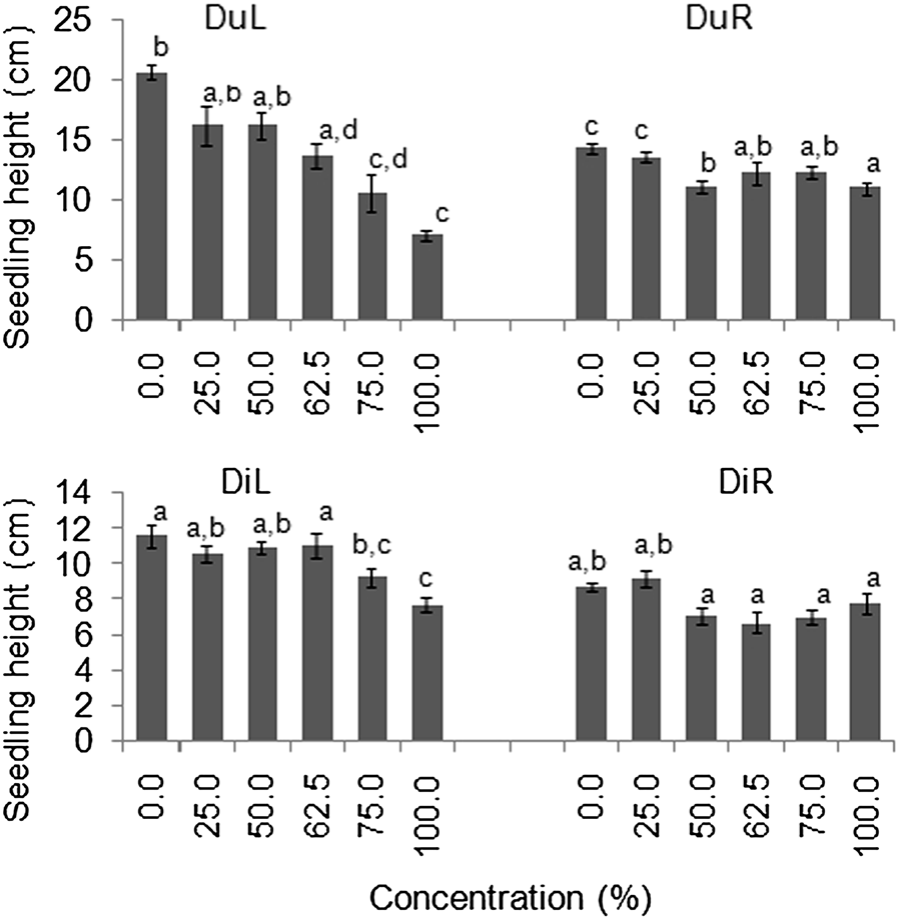
Mean (±S.E) seedling heights of germinated seeds in all groups after 30 days in laboratory experiment. *Bars* with *dissimilar letter(s)* are significant by Fisher LSD at *p* = 0.05; DuL (*D. uncinatum* leaf), DuR (*D. uncinatum* root), DiL (*D. intortum* leaf) and DiR (*D. intortum* root)

**Fig. 2:**
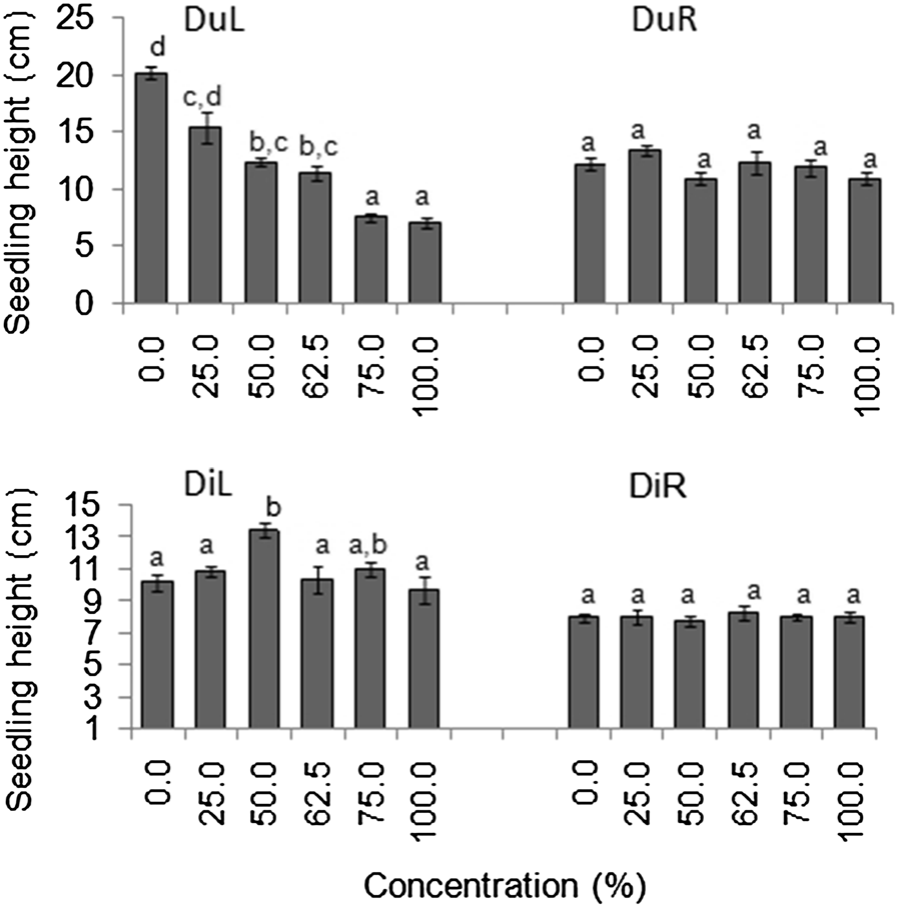
Mean (±S.E) seedling heights of germinated seeds in all groups after after 30 days in a screen house experiment. *Bars* with *dissimilar letter(s)* are significant by Fisher LSD at *p* = 0.05; DuL (*D. uncinatum* leaf), DuR (*D. uncinatum* root), DiL (*D. intortum* leaf) and DiR (*D. intortum* root)

Seedlings treated with higher concentrations (≥75 %) of DuL in both laboratory and screen house experiments were twice as short as those of higher concentrations of DuR, DiL and DiR (Figs. 1, 2).

**Fig. 3:**
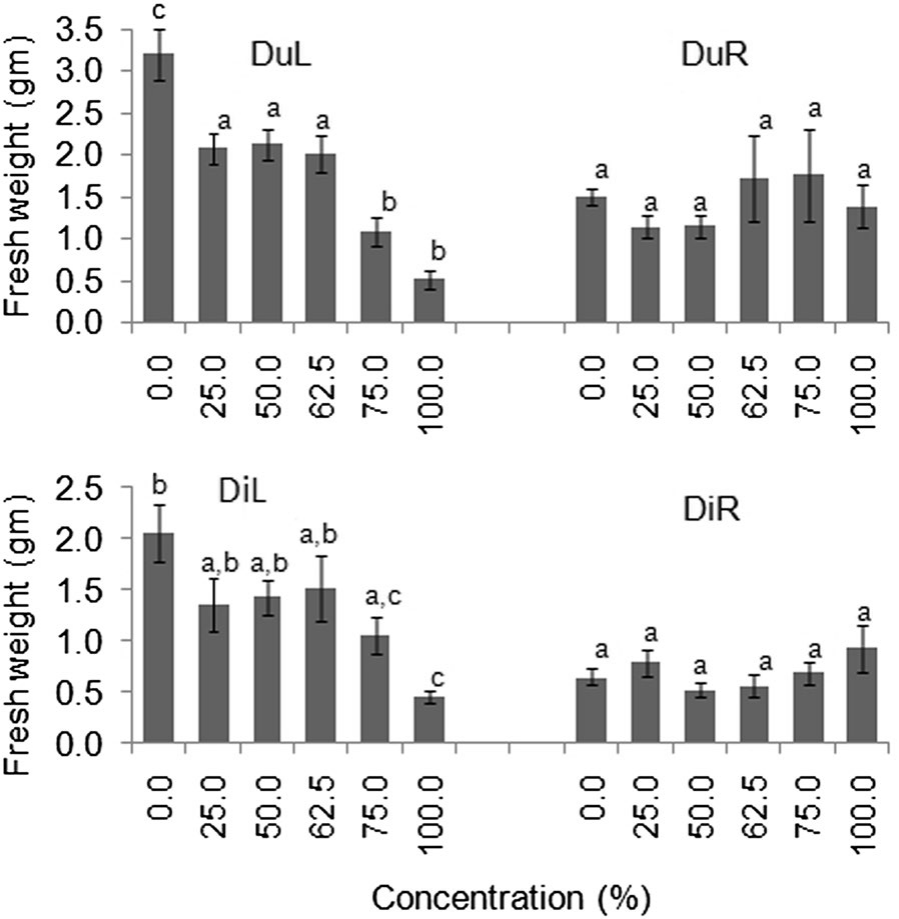
Mean (±S.E) seedling fresh weight of germinated seeds in all groups after 30 days in the laboratory experiment. *Bars* with *dissimilar letter(s)* are significant by Fisher LSD at *p* = 0.05; DuL (*D. uncinatum* leaf), DuR (*D. uncinatum* root), DiL (*D. intortum* leaf) and DiR (*D. intortum* root)

**Fig. 4:**
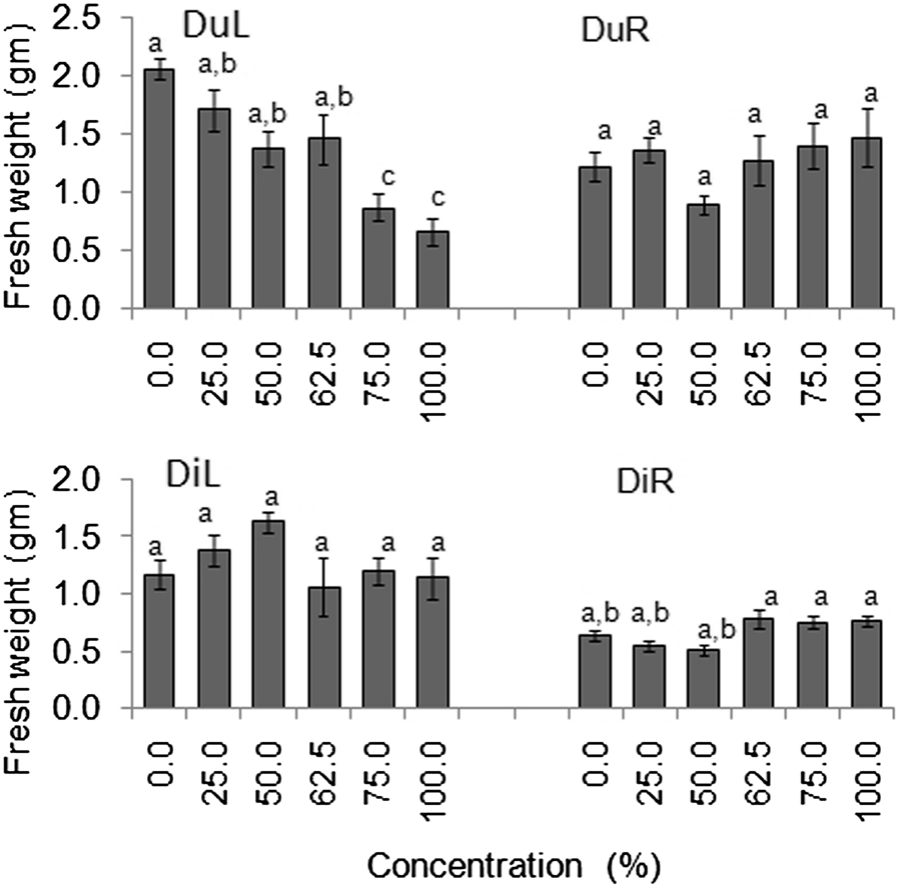
Mean (±S.E) seedling fresh weight of germinated seeds in all groups after 30 days in the screen house experiment. *Bars* with *dissimilar letter(s)* are significant by Fisher LSD at *p* = 0.05; DuL (*D. uncinatum* leaf), DuR (*D. uncinatum* root), DiL (*D. intortum* leaf) and DiR (*D. intortum* root)

DuL treatments had significant effects on seedling fresh weight in both laboratory and screen house experiments (*p* < 0.05) (Tables 4, 5). Seedlings treated with higher concentrations of DuL had three times lower fresh weight than those treated with lower concentrations (Figs. 3, 4).

**Fig. 5:**
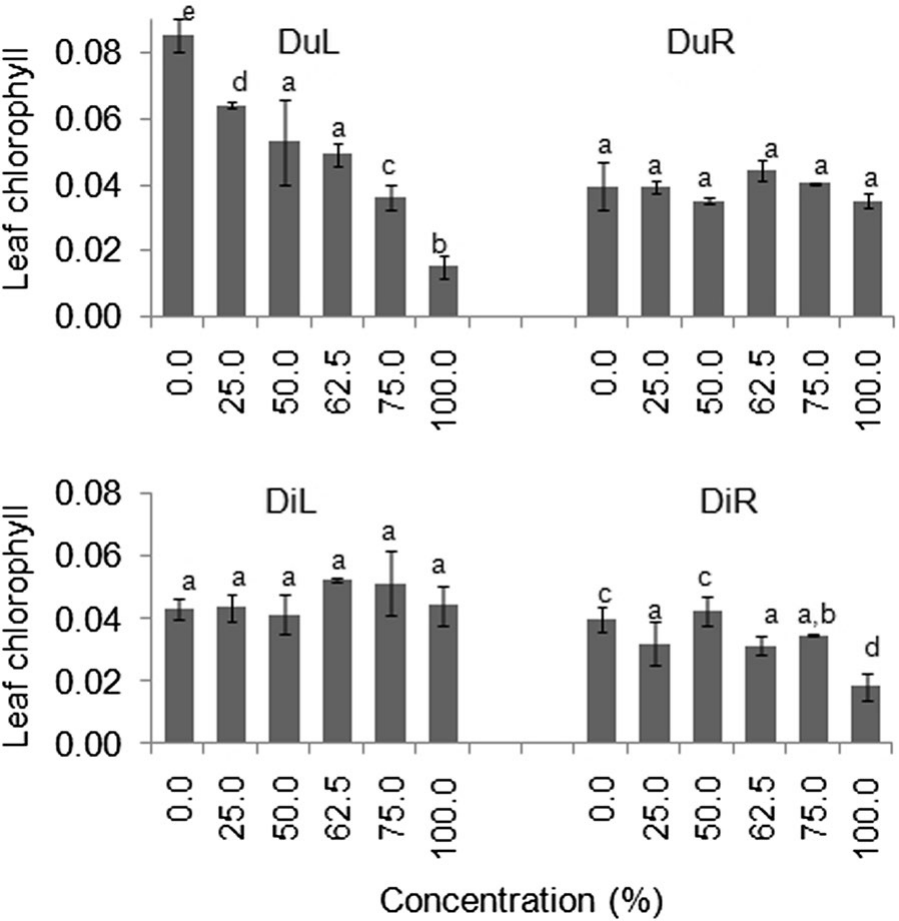
Mean (±S.E) seedling leaf total chlorophyll content of germinated seeds in all groups after 30 days in the laboratory experiment. *Bars* with *dissimilar letter(s)* are significant by Fisher LSD at *p* = 0.05; DuL (*D. uncinatum* leaf), DuR (*D. uncinatum* root), DiL (*D. intortum* leaf) and DiR (*D. intortum* root)

**Fig. 6:**
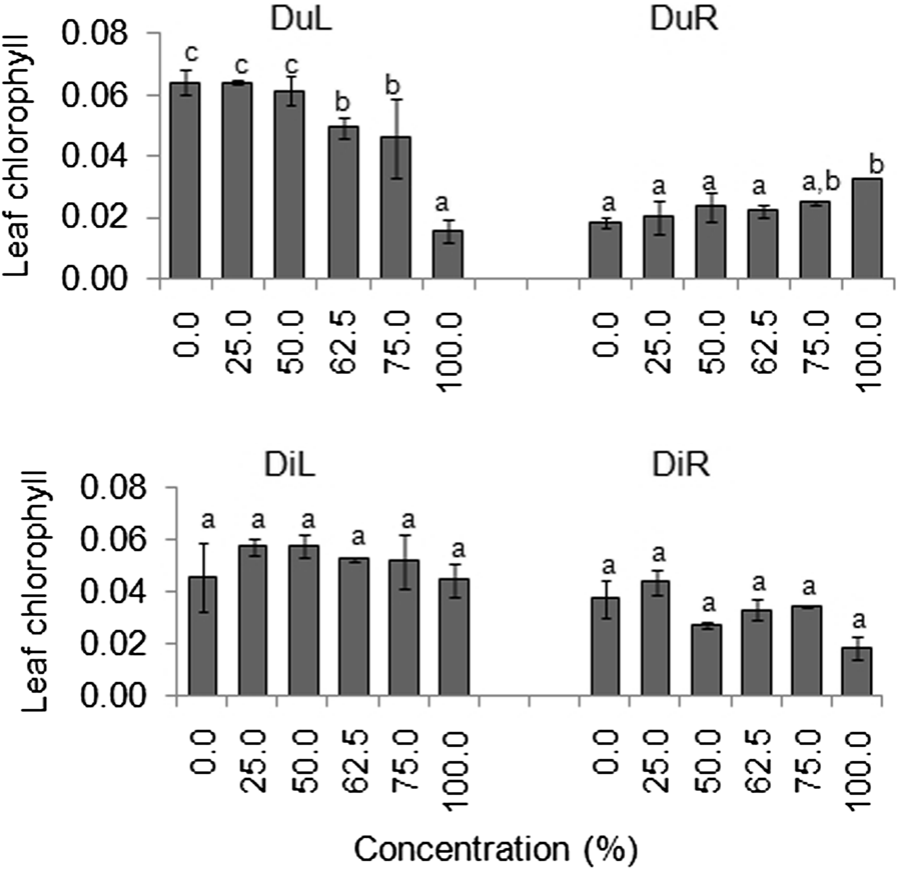
Mean (±S.E) seedling leaf total chlorophyll content of germinated seeds in all groups after 30 days in the screen house experiment. *Bars* with *dissimilar letter(s)* are significant by Fisher LSD at *p* = 0.05; DuL (*D. uncinatum* leaf), DuR (*D. uncinatum* root), DiL (*D. intortum* leaf) and DiR (*D. intortum* root)

Seedling leaf total chlorophyll (Chl) differed significantly across DuL treatments in both laboratory and screen house experiments (*p* < 0.05) (Tables 4, 5). Leaves of seedlings that were treated with higher concentrations of DuL had over three times lower Chl than those treated with lower concentrations (Figs. 5, 6).

In both laboratory and screen house experiments, a significant regression equation for the effects of extract on *G. cordifolia* seedling height, fresh weight and leaf chlorophyll content was only found for DuL (*p* < 0.05) (Table 6).

**Table 6:** Simple linear regression analysis results on the effects of DuL on *G. cordifolia* seedling height, fresh weight and leaf chlorophyll content after 30 days in laboratory and screen house experiments

Parameters	Laboratory					Screen house				
	*R* ^*2*^	*R*	*df*	*F*	*p*	*R* ^*2*^	*R*	*df*	*F*	*p*
Seedling height	0.59	−0.77	52	80.1	<0.001	0.78	−0.88	52	198.3	<0.001
Fresh weight	0.59	−0.77	52	79.1	<0.001	0.50	−0.71	52	55.2	<0.001
Chl	0.86	−0.93	52	346.6	<0.001	0.69	−0.83	52	120.3	<0.001

## Discussion

Although *G. cordifolia* is native to Tanzania, its invasiveness should not be ignored, as it supresses the native plants (pers. obs.). Further this species contains sesquiterpene lactone, which is an antimicrobial agent that might alter the overall metabolic functioning in ruminants (Amorim et al. 2013). We found that high treatment concentrations of DuL were effective in suppressing the germination and seedling vigor of *G. cordifolia* (Tables 1, 2). The efficiency of any herbicide depends mostly on its dosage (Khaliq et al. 2011). In some instances, there may be an unintentional overestimation of the dose (Zhang et al. 2000), which might cause serious ecological and environmental problems such as weed resistance and health hazards (Heap 2007). High concentrations of DuL extract suppressed the *G. cordifolia* germination rate by over 50 % compared to DuR, DiL and DiR. Similarly, the effectiveness of DuL in suppressing germination and seedling vigor of the invasive weed species *Tagetes minuta* was previously reported (Ngondya et al. 2016). Moreover, Khan et al. (2008) argued that the successful control of *Striga hermonthica* under maize field intercropped with *Desmodium* species was due to the strong allelopathic effects displayed by *D. uncinatum*. Therefore, *D. uncinatum* might probably have developed a competitive mechanism that accumulates high amounts of allelo-chemicals in its leaves, which inhibit germination of seeds of other plants (Khan et al. 2008). Therefore, when grown with other plants or as litter, this species may suppress and eventually outcompete other nearby growing plant species. Suppression of understorey plants by allelopathic litter as a competition strategy has previously been reported (McPherson and Thompson 1972). Allelopathic plants have further been successfully used for inhibiting the emergence of some weeds and pathogens in rice fields (Khanh et al. 2007). Interestingly, rice itself was reported to be allelopathic to both monocot and dicot weeds including *Echinochloa crusgalli*, an associated weed of paddy rice (Bhadoria 2011). This supports the idea for the potential of employing indigenous plants with allelopathy as a new source of natural herbicides and may help to reduce the present dependency for synthetic herbicides while at the same time aiding in the development of biological herbicides. Hence, the use of plants such as *D. uncinatum,* which are both allelopathic and with high agronomic (nutritive and palatable) values for ungulates, could be a better and effective alternative as a potential biological herbicide (Sodaeizadeh and Hosseini 2012).

Seedling vigor among other factors is represented by the seedling height (Islam et al. 2000; Taye et al. 2013). Taller plants ensure their leaves’ ability to intercept up to a recommended 95 % of the incoming solar radiation (Brougham 1956) and, hence, achieve an effective photosynthesis as a requirement for maximum growth. The benefit of taller plants in a community is an ensured access to light (Falster and Westoby 2003), although this competitive advantage depends on relative rather than absolute height. Shading of *Bougainville* species, for instance, increased flowering time, reduced number of flowers and leaf chlorophyll (Saifuddin et al. 2010). Therefore, taller plants will have stronger shoots compared to their counterparts as they invest much in stems and vascular structure for support, which in turn ensures their ability to withstand stresses such as animal trampling. Shorter plants, on the other hand, will suffer from a reduced number of flowers (fewer seed formation) which might result into limited dispersal to stunted growth due to reduced photosynthesis. We observed a significant decrease in seedling height with increasing extract concentrations with ≥75 % DuL being the most effective concentration. We think that this can be a milestone to the successful management of *G. cordifolia* based on our results showing reduced ability to perform photosynthesis and seedlings with weaker shoots that are susceptible to trampling by animals. In the long run, an emergence of native palatable plants such as *Cynodon dactylon, Chloris pycnothrix, Chloris gayana, Digitaria abysinica* and *Pennisetum clandestenum* that have been observed to co-exist with invasive weed *G. cordifolia* (Ngondya, unpublished data) might arise and, hence, provide sufficient food to herbivores.

We further observed that higher DuL concentration successfully suppressed seedling fresh weight, which might render them more susceptible to pathogens as well as abiotic stresses (Krishnamurthy et al. 2011). We, therefore, propose that affecting seedling stability by suppressing its fresh weight will be an added advantage to the management of the undesired invasive weeds such as *G. cordifolia*. As deposition of seeds by animals normally occurs in areas where they spend most of their time (Dennis et al. 2007) and as these areas will be more trampled, spraying of ≥75 % DuL extract will affect *G. cordifolia* seedlings fresh weight and, hence, make them more susceptible to mechanical damage, which will additionally reduce this species’ abundance.

As we expected, total leaf chlorophyll contents decreased with increasing treatment concentration, particularly under DuL treatments. Plant growth, development and adaptation to various different environmental conditions depends strongly on its leaf’s photosynthetic efficiency, associated with chlorophyll content (Beltramin da Fonseca et al. 2013). Leaf chlorophyll, therefore, is essential in the conversion of the solar radiation into chemical energy (ATP and NADPH) and, thus, for plant growth and development (Araujo et al. 2013). While leaves that have higher chlorophyll show a better photosynthesis performance than their counterparts (Gabrielsen 1948), those with low chlorophyll content have been associated with low competitive ability for light and, thus, to survive (Krause and Weis 1991). Moreover, plants with reduced leaf chlorophyll content are likely to produce flowers with accelerated abscission (Saifuddin et al. 2010), which might reduce nectare availability and consequently low seed dispersal through pollinators. We, therefore, speculate that affecting *G. cordifolia* seedling chlorophyll content with higher concentration of DuL (≥75 %) will add to the effort towards successful management of this invasive weed plant.

While the four discussed parameters in most weeds can be efficiently suppressed through chemical herbicides (Mahmood et al. 2013; CDFA 2014) we suggest a novel way of using natural components leading to the same effect but with less harm to the environment. The spraying of extracts as biological herbicides has proved to be a successful weed management tool without affecting productivity in cotton, soybean, wheat and rice (Soltys et al. 2013). Based on our results, we are expecting a suppression of more than 70 % of *G. cordifolia* seedlings in affected areas with a single spray application of 100 ml (≥75 %) of *D. uncinatum* leaf extract per 0.03 m^2^. Currently, timed-mowing of *G. cordifolia* before anthesis and controlled burning are among the management options that are utilized in Ngorongoro Crater (pers. obs.). These two strategies are only shortterm solutions. Longterm sustainable solutions such as the use of *D. uncinatum* extract that not only affect individual *G. cordifolia* but also reduces its soil seedbank and, hence, reduces its chance of germinating in the future, are therefore highly recommended. Moreover, *D. uncinatum* is readily available as it can be grown easily, thereby providing a possibility for future development of a biological herbicide that can help in invasive species management.

## Conclusion

This study has shown that a natural extract can offer remedies to the negative impacts of invasive plant species in rangelands and is especially applicable in areas sensitive/limited to the use of synthetic herbicides for sustainable rangeland management. Based on our findings, spraying the landscape infested with seeds or seedlings of *G. cordifolia* with approximately 100 ml of ≥75 % DuL extract per 0.03 m^2^ will keep the abundance of this non-palatable invasive plant low. This treatment will limit *G. cordifolia* growth and prevent its future spread within NCA. In the long run, using a biological herbicide might provide a beneficial management approach to suppress an aggressive invasive species such as *G. cordifolia*, which might invade various rangelands inside and outside of most protected areas. We suggest that further research is needed to identify the mechanisms responsible for *Desmodium spp* in reducing the germination and growth of *G. cordifolia*.

## Abbreviations

LITI: Livestock Training Institute
DuL: *Desmodium uncinatum* leaf
DuR: *Desmodium uncinatum* root
DiL: *Desmodium intortum* leaf
DiR: *Desmodium intortum* root
NCA: Ngorongoro Conservation Area
Chl: leaf total chlorophyll content
UNESCO: The United Nations Educational, Scientific and Cultural Organization
DMSO: dimethyl sulfoxide
ANOVA: analysis of variance
LSD: least significant difference
S.E: standard error
ATP: adenosine tri phosphate
NADPH: nicotinamide adenine dinucleotide phosphate hydrogen
CDFA: California Department of Food and Agriculture
GoT: Government of Tanzania
NM-AIST: Nelson Mandela African Institution of Science and Technology
